# Marked Elevation in Plasma Osteoprotegerin Constitutes an Early and Consistent Feature of Cerebral Malaria

**DOI:** 10.1160/TH15-10-0796

**Published:** 2016-01-14

**Authors:** Niamh O’Regan, Chris Moxon, Kristina Gegenbauer, Jamie M. O’Sullivan, Alain Chion, Owen P. Smith, Roger J. S. Preston, Teresa M. Brophy, Alister G. Craig, James S. O’Donnell

**Affiliations:** 1 Haemostasis Research Group, Institute of Molecular Medicine, Trinity Centre for Health Sciences, St James’s Hospital, Trinity College Dublin, Ireland; 2 Institute of Infection and Global Health, University of Liverpool, UK; 3 Malawi-Liverpool-Wellcome Trust Clinical Research Programme, University of Malawi College of Medicine, Blantyre, Malawi; 4 National Children’s Research Centre, Our Lady’s Children’s Hospital, Crumlin, Dublin, Ireland; 5 Haematology Dept, Our Lady’s Children’s Hospital, Dublin, Ireland; 6 Department of Clinical Medicine, School of Medicine, Trinity College Dublin, Ireland; 7 Department of Parasitology, Liverpool School of Tropical Medicine, Liverpool, UK; 8 National Centre for Hereditary Coagulation Disorders, St James’s Hospital, Dublin, Ireland

**Keywords:** Osteoprotegerin, von Willebrand factor, cerebral malaria, *Plasmodium falciparum*, *Plasmodium berghei*

## Abstract

Adherence of infected erythrocytes to vascular endothelium causes acute endothelial cell (EC) activation during *Plasmodium falciparum* infection. Consequently, proteins stored in Weibel-Palade (WP) bodies within EC are secreted into the plasma. Osteoprotegerin (OPG) binds to VWF and consequently is stored within WP bodies. Given the critical role of EC activation in the pathogenesis of severe malaria, we investigated plasma OPG levels in children with *P. falciparum* malaria. At presentation, plasma OPG levels were significantly elevated in children with cerebral malaria (CM) compared to healthy controls (means 16.0 vs 0.8 ng/ml; p<0.01). Importantly, OPG levels were also significantly higher in children with CM who had a fatal outcome, compared to children with CM who survived. Finally, in children with CM, plasma OPG levels correlated with other established prognostic indices (including plasma lactate levels and peripheral parasite density). To further investigate the relationship between severe malaria and OPG, we utilised a murine model of experimental CM in which C57BL/6J mice were infected with *P. berghei* ANKA. Interestingly, plasma OPG levels were increased 4.6 fold within 24 hours following *P. berghei* inoculation. This early marked elevation in OPG levels was observed before any objective clinical signs were apparent, and preceded the development of peripheral blood parasitaemia. As the mice became increasingly unwell, plasma OPG levels progressively increased. Collectively, these data suggest that OPG constitutes a novel biomarker with prognostic significance in patients with severe malaria. In addition, further studies are required to determine whether OPG plays a role in modulating malaria pathogenesis.

## Introduction

*Plasmodium falciparum* malaria continues to be associated with significant morbidity and mortality (1, 2). In particular cerebral malaria (CM), a severe encephalopathy caused by *P. falciparum* infection, has a mortality of 10–20% and a high rate of neurological sequelae in survivors (3). Although the molecular mechanisms underlying the pathogenesis of CM remain poorly understood, previous studies have demonstrated a critical role for endothelial cell (EC) activation (4, 5). In particular, a number of specific EC surface receptors, including CD36, endothelial protein C receptor (EPCR), E-selectin and intercellular adhesion molecule-1 (ICAM-1), have been shown to regulate the adhesion and sequestration of *P. falciparum-*infected erythrocytes (IE) within the microvasculature (6). Quantitative expression of these different EC surface receptors on EC surfaces is modified by inflammatory cytokines, such as interleukin-1 and tumour necrosis factor (7). In addition to these IE surface receptors, EC also contain intracellular storage organelles known as Weibel-Palade (WP) bodies (8). These WP bodies are rod-shaped organelles that can be up to 5 µm in length and are specific to EC. von Willebrand factor (VWF) synthesis within EC is a prerequisite for WP body formation (9, 10). In normal plasma, VWF circulates as a series of heterogeneous multimers. In contrast, VWF stored within WP bodies is enriched in high-molecular-weight multimers (HMWM) that demonstrate enhanced binding affinities for both collagen and platelets (8, 10). In addition, a number of other EC proteins have also been identified as WP body constituents. These include VWF propeptide (VWFpp), P-selectin, factor VIII, angiopoietin-2, tissue-type plasminogen activator (tPA), interleukin-8, galectin-3 and osteoprotegerin (OPG) (8, 10). Following acute EC activation, WP bodies fuse with the EC surface membrane, and their contents are secreted directly into the plasma.

Previous studies from our laboratory and others have demonstrated that plasma levels of HMWM VWF and VWFpp are both markedly elevated in patients with severe *P. falciparum* malaria (11–17). Furthermore, plasma VWF:Ag and VWFpp levels have also been shown to correlate with other biochemical prognostic markers in patients with severe malaria (14, 18). Interestingly, in a study of healthy volunteers infected with *P. falciparum*, de Mast et al. observed that the increase in plasma VWF:Ag and VWFpp levels was evident from a very early stage following *P. falciparum* infection (19). The molecular mechanism(s) underlying this early marked EC activation and WP body secretion have not been defined. Nevertheless, emerging data suggest that the early release of WP body contents, and in particular VWF, may play a role in malaria pathogenesis. We recently reported that platelet-decorated HMWM VWF strings on the surface of activated EC can directly adhere to trophozoite-stage *P. falciparum* IE in a shear-based assay (20). Thus, the rapid release of HMWM VWF multimers from WP bodies, which occurs at an early stage in the clinical course of *P. falciparum* infection, may provide a novel mechanism through which IE can adhere to activated EC surfaces.

In keeping with the marked elevated VWF and VWFpp levels observed in children with cerebral malaria (CM), plasma levels of Angiopoietin-2 (Ang-2), another protein stored within WP bodies, are also significantly elevated (11, 13, 21, 22). Moreover, higher plasma Ang-2 levels have also been associated with a worse clinical outcome (21–23). Osteoprotegrin (OPG) is another EC protein stored within WP bodies (24). OPG is recruited into WP bodies by binding directly to the A1 domain of VWF (24, 25). OPG plays a key role in regulating bone remodelling by acting as a soluble decoy receptor for Receptor Activator of NFκB Ligand (RANKL) expressed on osteoblasts (26). Recent studies have also identified functional roles for OPG in modulating vascular biology (27, 28). In particular, OPG has been shown to upregulate expression of adhesion molecules on EC surfaces, and thereby facilitate leucocyte adhesion (29, 30). Furthermore, OPG influences EC apoptosis, and elevated OPG levels have been associated with vascular disease (27, 28). Given the key role of EC activation in the pathogenesis of severe *P. falciparum* malaria, we have investigated plasma OPG levels in children with cerebral malaria. To further understand the relationship between WP body secretion and malaria, we have investigated OPG levels in an established murine model of experimental CM.

## Materials and methods

### Patient samples

Children aged 6 months to 12 years were recruited at Queen Elizabeth Central Hospital, Blantyre, Malawi between 2008 and 2011. Children were recruited into one of four prospectively defined groups: 1) CM, defined as Blantyre coma score ≤2, peripheral *P. falciparum* parasitaemia and an absence of clinical evidence for another cause of coma; 2) uncomplicated malaria, defined as an acute febrile illness in a child with peripheral *P. falciparium* parasitaemia and no evidence of organ compromise; 3) non-malarial febrile illness, defined acute febrile illness in a aparasitaemic child with no evidence of organ compromise and; 4) healthy controls, recruited from otherwise well children having elective surgery. We screened for parasitaemia using a rapid diagnostic test that employs a *P. falicaprum* specific antigen (First Response, Premier Medical, Kachigam, India) and if positive, peripheral parasite density was confirmed and quantified by thick and thin blood smears, as described previously (31). We have published data examining coagulation factors and soluble receptors in this cohort previously (31, 32), but the data presented here on VWF and OPG levels is presented for the first time. The study was approved by the ethical boards at the College of Medicine in Malawi (no. P.02/ 10/860) and Liverpool School of Tropical Medicine in the United Kingdom (no. 09.74). Informed consent was obtained from the parents or legal guardians of all the children enrolled in this study in accordance with the Declaration of Helsinki.

### Murine studies

Previous studies have demonstrated that wild type (WT) C57BL/6 mice infected with *P. berghei* ANKA develop a complex neurological syndrome that includes many similar clinical features (including paralysis, seizures and coma) to those observed in patients with CM (33–35). Depending upon the *P. berghei* inoculation dose, mice typically die within 6 to 10 days from this experimental CM. Consequently we used this murine model of experimental CM to further investigate the relationship between severe malaria and OPG. All mouse experiments were performed in compliance with Irish Medicines Board regulations, and were reviewed and approved by the Trinity College Dublin BioResources Ethical Committee. WT C57BL/6J mice were bred in-house under standard pathogen-free conditions, and all experiments were performed on mice aged 8-10 weeks. Mice were infected by intraperitoneal injection of 2×10^6^ *P. berghei* ANKA. Following inoculation, the mice were monitored using a previously validated clinical scoring system to assess progression of experimental CM (36). In addition, peripheral blood *P. berghei* parasitaemia levels were monitored daily by examination of Giemsa-stained thin blood smears obtained from tail vein bleeds. Blood samples at predefined time points following *P. berghei* infection were also collected by cardiac puncture into acid citrate dextrose (ACD) anticoagulant (85 mM trisodium citrate, 65 mM citric acid, 100 mM glucose) (Sigma, Arklow, Ireland). Platelet-poor plasma was prepared by centrifugation of blood samples at 1500 g for 15 minutes at 20 °C. The samples were then aliquoted and stored at –80 °C until use.

### Determination of plasma VWF:Ag and OPG levels

Human plasma VWF:Ag levels were measured using a previously described enzyme-linked immunosorbent assay (ELISA) (37). In brief, Maxisorp plates (Nunc, Roskilde, Denmark) were coated with rabbit anti-human VWF antibody (Dako, Glostrup, Denmark) in 50 mM sodium carbonate buffer. After blocking with 3% bovine serum albumin (Sigma) test samples or reference plasma were then added at appropriate dilutions. Bound VWF was subsequently detected using horseradish peroxidase–conjugated (HRP) rabbit anti-human VWF antibody (Dako). Following further washing, HRP substrate 3,3’,5,5’-Tetramethylbenzidine (TMB; Substrate Reagent Pack, R&D Systems, Abingdon, UK) was then added, and the reaction finally terminated by addition of 1 M H_2_SO_4_. Absorbance was read at 450 nM using a VERSAmax microplate reader (Molecular Devices, Winnersh, UK). Human and murine plasma OPG levels were determined using commercial OPG ELISAs (Osteoprotegerin/TNFRSF11B DuoSet^®^, R&D Systems) performed in accordance with the manufacturer’s guidelines.

### Statistical analysis

All experimental data and statistical analysis were performed using the GraphPad Prism program (Graphpad Prism version 5.0 for Windows; GraphPad Software, Inc. San Diego, CA, USA) and statistical significance was assigned at a value of p<0.05. Normally distributed data were expressed as mean values ± standard error of the mean (SEM). To assess statistical differences, data were analysed using Student’s unpaired two-tailed t-test. The Spearman correlation coefficient was used to determine linear association between different variables.

## Results

### Clinical characteristics

A total of 135 children with cerebral malaria, 45 children with non-malarial febrile illness, 52 children with uncomplicated malaria and 26 healthy controls were enrolled in this study. Baseline clinical and laboratory characteristics are summarised in ► Table 1.

### Plasma VWF:Ag and OPG levels are markedly elevated in children with CM

**Figure 1:**
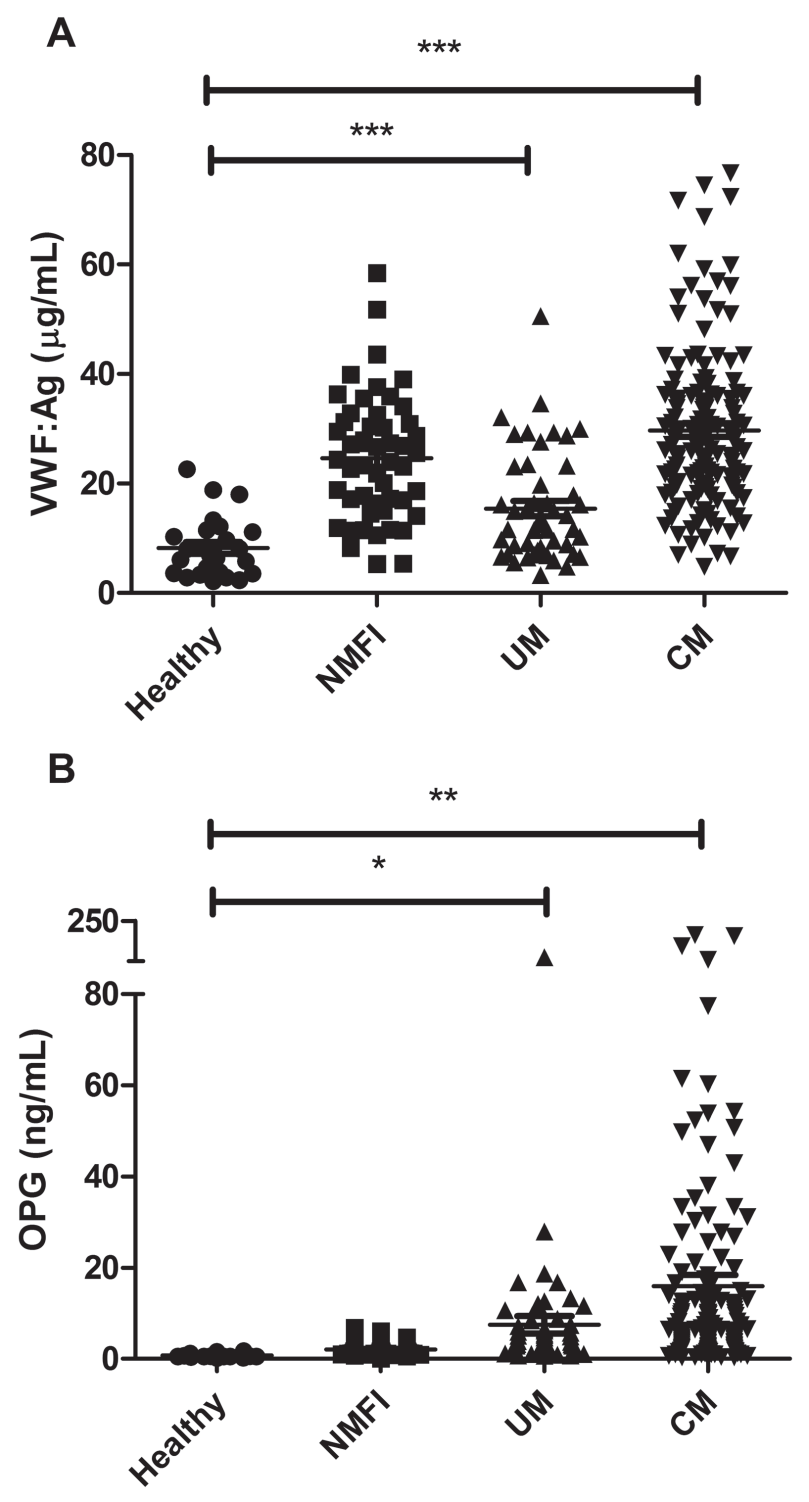
**Plasma VWF:Ag and OPG are markedly elevated in children with CM.** Plasma VWF:Ag (A) and OPG levels (B) were determined by ELISA in children presenting with cerebral malaria (CM), uncomplicated malaria (UM), or non-malarial febrile illness (NMFI) as well as in a cohort of healthy control children. Each plasma sample was tested in duplicate at three dilutions, and mean values for each group are shown. VWF:Ag and OPG levels were significantly elevated in children with CM compared to controls. An unpaired Student’s t-test was performed for statistical analysis and significance is indicated as follows: * P<0.05, ** P<0.01, *** P<0.0001, respectively.

At presentation, plasma VWF:Ag levels were significantly elevated in Malawian children with CM compared to healthy control children (means 29.7 vs 8.3 µg/ml; p<0.001) (►Figure 1A). This ~3.6 fold increase is similar in magnitude to that previously reported in other cohorts of paediatric and adult patients with severe *P. falciparum* infection (12, 14–16). Plasma VWF:Ag levels were also mildly increased in children with uncomplicated malaria (UM). However the elevation in plasma VWF:Ag observed in children with UM was similar to that observed in children with other non-malarial febrile illnesses (NMFI). Plasma OPG levels were also markedly elevated in children with CM compared to healthy controls (means 16.0 vs 0.79 ng/ml; p<0.01) (► Figure 1 B). However in contrast to the 3.6-fold increase observed in VWF:Ag levels, plasma OPG levels were increased more than 20-fold. Interestingly, in children with CM, plasma OPG levels varied over a wide range, which may reflect that individal children were presenting at different times in the progression of their malaria.Plasma OPG levels were also significantly elevated in children with UM compared to healthy controls (p<0.05). Although plasma VWF:Ag levels were significantly elevated in children with NMFI, plasma OPG levels were not significantly increased in this cohort. Thus, the marked increase in OPG seems to constitute a relatively malaria-sensitive phenomenon. Collectively, these findings support the hypothesis that acute endothelial cell activation and WP body secretion constitute characteristic features of severe *P. falciparum* malaria. In addition, these data further demonstrate for the first time that plasma OPG levels are markedly elevated in children with *P. falciparum*.

### Plasma OPG levels relate to malarial severity in children with *P. falciparum* malaria

**Figure 2:**
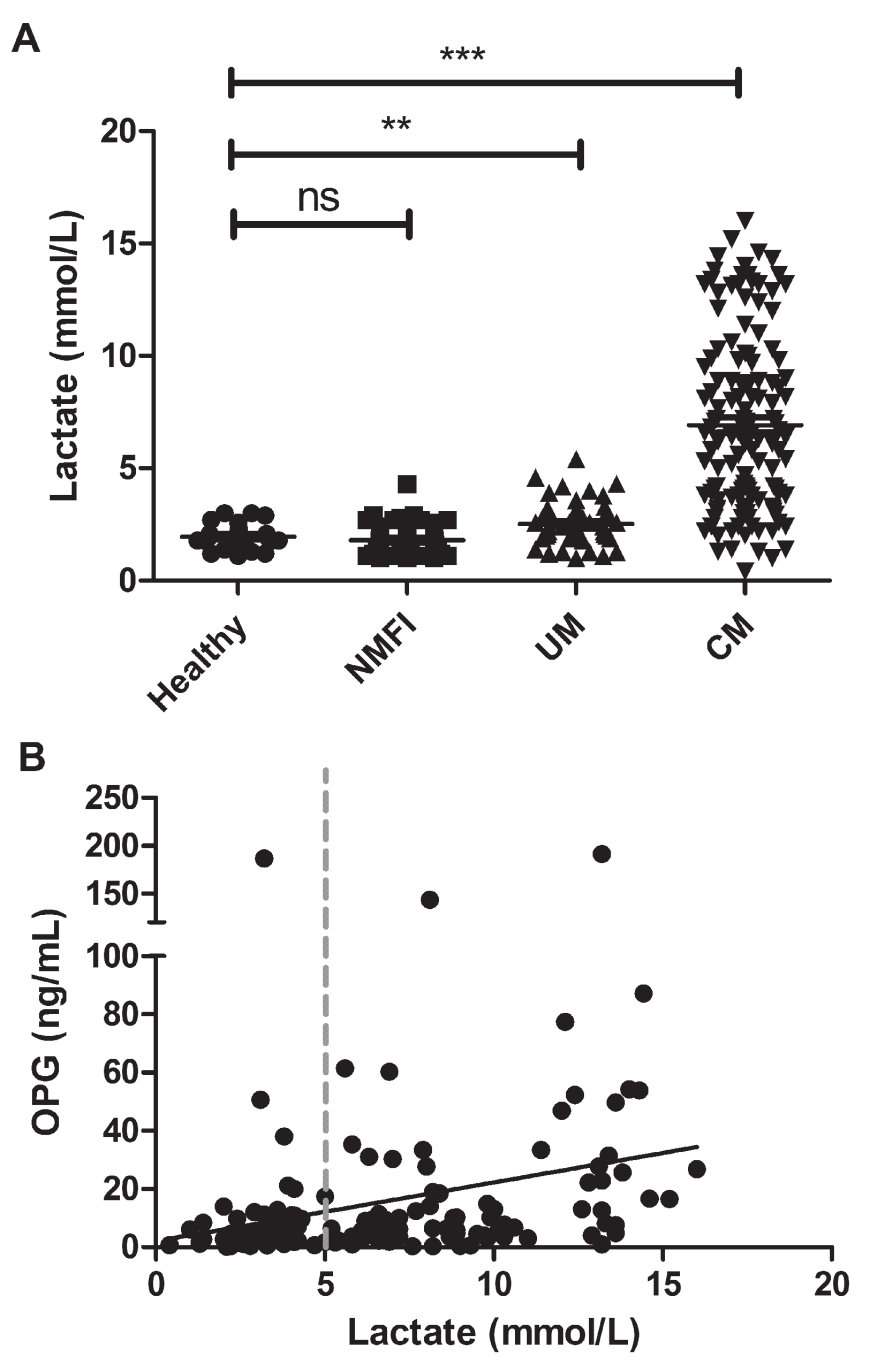
**Relationship between plasma OPG levels and lactate levels in children with CM.** A) Plasma lactate levels were performed by ELISA in children with CM, UM, NMFI and healthy controls. (** P<0.01, *** P<0.0001, n.s. = not significant). B) Relationship between plasma OPG levels and lactate levels in children with CM. The dashed red line at 5 mmol/l defines plasma lactate level considered diagnostic of lactic acidosis.

**Figure 3:**
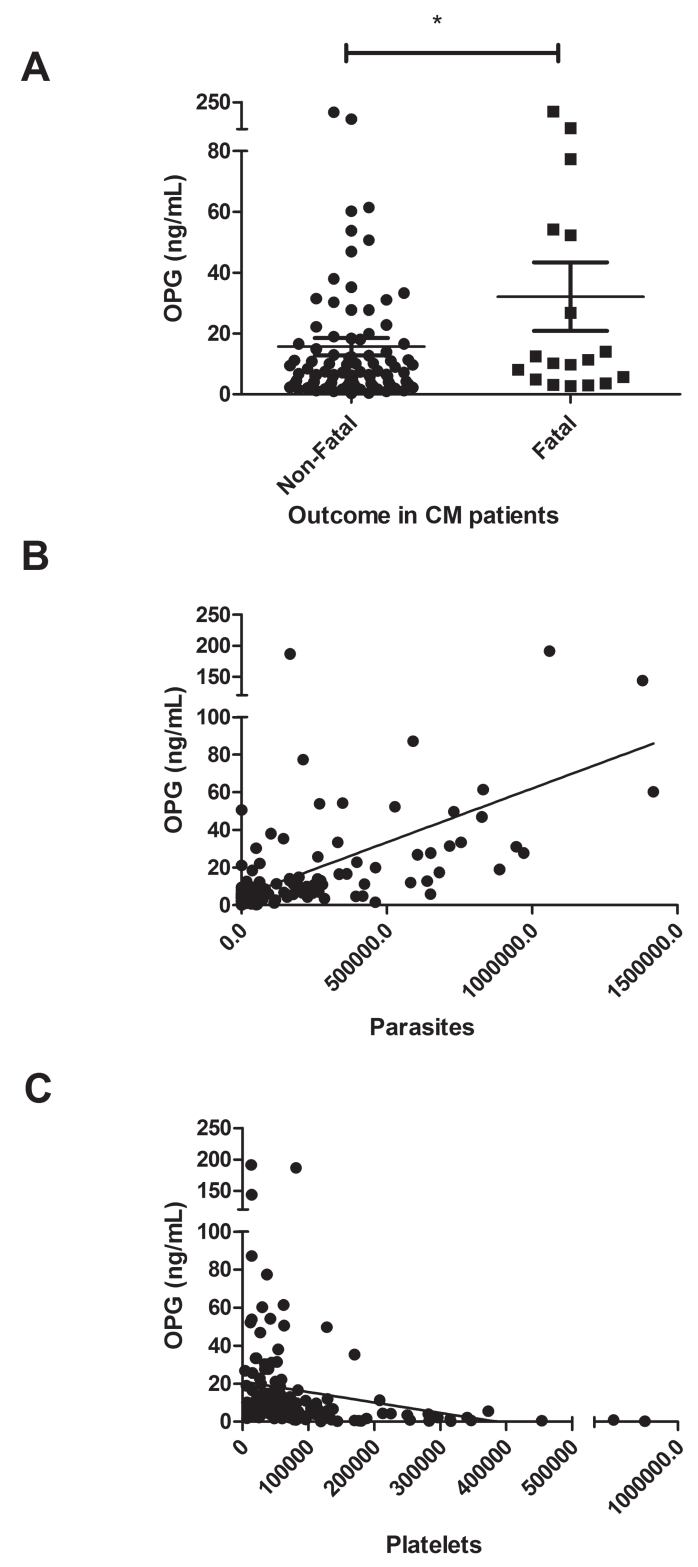
**Plasma OPG levels relate to malaria severity in children with *P. falciparum* malaria.** A) Plasma OPG levels at presentation in children with CM and a fatal outcome were compared to those of children with CM and a non-fatal outcome. B) Plasma OPG levels in children with CM correlated with peripheral blood *P. falciparum* levels. C) Plasma OPG levels inversely correlated with platelet count in children with CM.

In patients with severe *P. falciparum* malaria, it is well established that plasma lactate levels constitute a useful biochemical prognostic indicator (38). Unsurprisingly, plasma lactate levels were significantly elevated in children with CM compared to healthy controls (means 6.9 vs 2.0 mmol/l; p<0.001) (►Figure 2A). In addition, plasma lactate levels were significantly higher in children with CM compared to those with UM respectively (p<0.001). Among the 135 children with confirmed CM, plasma OPG levels and lactate levels were significantly correlated (r=0.42, p<0.001) (►Figure 2B). Eighty-four children with CM demonstrated plasma lactate levels greater than 5 mmol/l consistent with lactic acidosis. Plasma OPG levels in children with CM and lactic acidosis were higher compared to levels in children with CM uncomplicated by lactic acidosis (means 19.6 vs 10.2; p=0.06). Importantly, plasma OPG levels at admission were also significantly higher in the 21 children with CM who later died, than in the 114 children with CM who survived (means 29.6 vs 14 ng/ml; p=0.026) (►Figure 3A). Peripheral parasite density and platelet count have previously been defined as markers of disease severity in patients with severe *P. falciparum*. In previous studies, we found no significant correlation between plasma VWFpp levels and peripheral parasite density (14, 15). In contrast, in children with CM we observed an association between OPG levels and parasite density (r=0.65; p<0.001) (►Figure 3B). OPG levels were also negatively associated with platelet count (r= –0.5; p<0.001) (►Figure 3C).

### Plasma OPG levels are markedly elevated in experimental CM

**Figure 4:**
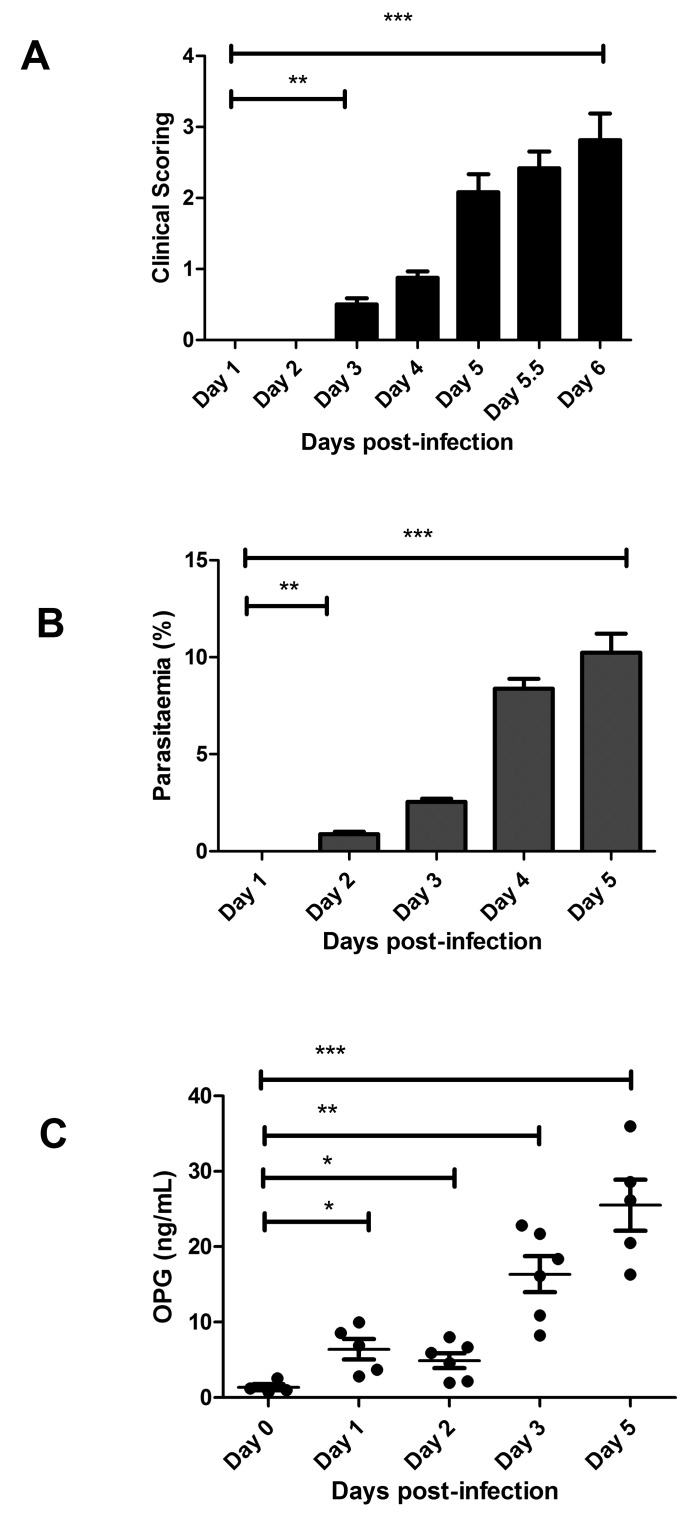
**Marked elevation in plasma OPG levels constitutes an early and characteristic feature of experimental CM.** WT C57BL/6J mice (n=12) mice were inoculated with 2 ×10^6^ *P. berghei* ANKA parasites. A) Clinical progression of the malaria infection was assessed were compared using a validated clinical scoring algorithm. Results presented illustrated represent the mean values ± SEM unless otherwise stated.B) Following infection with *P. berghei*, peripheral blood parasitaemia levels were determined on a daily basis from Giemsa-stained smears. C) Whole blood samples were collected from infected mice by cardiac puncture, and plasma OPG levels were then measured at pre-defined time points using ELISA. All ELISAs were performed in triplicate, and results presented represent the mean values ± SEM (* P<0.05, ** P<0.01, *** P<0.0001, respectively).

To further investigate the relationship between severe malaria and OPG, we utilised an established murine model of experimental CM. In this model, C57BL/6J mice were infected with *P. berghei* ANKA. Malaria progression was then assessed using a previously validated clinical scoring algorithm (36). Using this algorithm, overt clinical signs of malaria were first observed on Day +3 following *P. berghei* infection. Subsequently, the infected mice became progressively unwell, with death typically occurring 6–7 days after infection (►Figure 4A). Peripheral blood smear analysis demonstrated that *P. berghei* parasitaemia levels were first detectable 48 hours (h) following inoculation (►Figure 4B). Thereafter, blood parasitaemia levels progressively increased as the malaria infection progressed. In keeping with our findings in children with CM, a significant increase in plasma OPG levels was also observed in wild-type C57BL/6J mice following *P. berghei* infection (►Figure 4C). For example, by Day +5 following inoculation, mean murine plasma OPG levels were increased approximately 18 fold (1.38 ng/ml at Day 0 vs 25.5 ng/ml at Day+5; p<0.001). This increase in murine plasma OPG levels seen was thus strikingly similar in magnitude to that observed in children with *P. falciparum* CM.

Interestingly, a significant increase in plasma OPG levels was observed from a very early stage following *P. berghei* infection. Plasma OPG levels were already increased 4.6 fold (1.38 vs 6.4 ng/ml; p<0.05) within 24 h following *P. berghei* inoculation. This early marked elevation in murine plasma OPG levels was established before any objective clinical signs were apparent (►Figure 4A). Furthermore, the increase in plasma OPG levels also preceded the development of significant peripheral blood parasitaemia levels (►Figure 4B). As the mice became increasingly unwell, plasma OPG levels progressively increased. Finally, and again in keeping with our observations in children with CM, a correlation between plasma OPG levels and peripheral blood parasitaemia levels was also observed in the murine ECM model (r=-0.9; p=0.08). Collectively, these findings confirm that marked elevation in plasma OPG constitutes a consistent finding in both human and experimental murine CM. Moreover, the increase in plasma OPG occurs at an early stage during the pathogenesis of severe malaria infection.

## Discussion

EC activation plays a critical role in modulating the pathogenesis underlying severe *P. falciparum* malaria (4, 5). This EC activation triggers the secretion of WP body contents into the plasma. Previous studies have consistently shown that plasma levels of WP body-stored HMWM VWF are significantly increased in patients with both *P. falciparum* and *P. vivax* malaria (12, 14–16). Furthermore, significant elevations in the plasma levels of other WP components (including VWFpp and Ang-2) have also been previously reported in patients with malaria infection (13, 14, 21). In this study, we demonstrate for the first time that severe *P. falciparum* malaria is also associated with a marked increase in plasma OPG levels. Although the biological mechanism(s) responsible for this increase in OPG have not been defined, it seems likely that fulminant acute endothelial cell activation is at least in part responsible. Interestingly, we observed a similar marked increase in plasma OPG levels in a murine model of experimental CM. In addition, using this murine model we demonstrated that the increase in plasma OPG develops at an early stage following malaria infection. Indeed, plasma OPG levels were significantly increased before parasitaemia was detectible, and well before the onset of any clinical signs. This finding is consistent with previous studies in which elevations in plasma VWF levels were observed at a surprisingly early stage in both human volunteers infected with *P. falciparum*, and in mice infected with *P. berghei* (19)*.*

The early and marked increase in plasma OPG levels associated with malaria may represent an epiphenomenon, consistent with acute EC activation and WP body secretion. Alternatively, it is possible that OPG may have a role in the pathogenesis of severe malaria. Although OPG has an established role in regulating osteoclast differentiation, more recent studies have identified additional novel biological functions. Of particular relevance, OPG has been shown to influence the expression of adhesion molecules on the EC surface, and to modulate EC apoptosis (28–30). For example, i*n vitro* studies have shown that OPG increases expression of Ang-2, VCAM-1, and ICAM1 on HUVEC (29). Interestingly, in this study we found significant correlations between plasma OPG levels and several other prognostic markers (including plasma lactate levels, parasitaemia and thrombocytopenia) in children with CM. Further studies will be required to determine whether OPG plays any direct role in contributing to malaria pathogenesis.

Notwithstanding any putative role of OPG in malaria pathogenesis, our combined human and animal data support the hypothesis that plasma OPG levels may constitute a novel biomarker in malaria. However, the clinical utility of changes in plasma OPG levels as a measure of malaria severity, or indeed as a useful diagnostic or prognostic clinical tool, remains to be established. In this context, it is interesting that although both VWF and OPG are stored together within WP bodies, the increase in plasma OPG levels in children with CM was much more marked than that in VWF levels. This discrepancy may relate to different relative concentrations of VWF and OPG being stored within WP bodies. Alternatively, it is not known whether significant differences in plasma half-life may exist between OPG and VWF. Finally, significant OPG synthesis has also been described in other non-EC tissues (e.g. smooth muscle cells) (39). Consequently, although WP secretion is likely to be important in the aetiology of the markedly elevated plasma OPG levels in severe malaria, our findings do not preclude the possibility that the increased plasma OPG may also be in part derived from other cells. Additional studies to define the cellular origins of the markedly elevated plasma OPG levels will therefore be of interest.

The combined increases in plasma levels of both VWF and OPG observed in severe malaria are of further interest in that previous studies have demonstrated that OPG can bind directly to the A1 domain of VWF (24, 25). This OPG-VWF binding occurs within EC prior to secretion from WP bodies (25). Due to the ionic conditions existing in normal plasma, it seems unlikely that any further OPG binding to VWF takes place in the peripheral circulation. Nevertheless, VWF-OPG complexes formed prior to secretion from WP bodies have been shown to remain relatively stable in normal plasma (25). Further studies will be required to determine whether increased levels of this VWF-OPG complex are circulating in patients with severe malaria. Moreover, it remains unclear whether the formation of this complex has any effect upon the normal functional properties of either VWF and/or OPG, respectively.

In conclusion, we have shown that plasma OPG levels are elevated approximately 20-fold in both children with CM, and in a murine model of experimental CM. This marked increase in plasma OPG is evident from an early stage following malaria infection. Furthermore, plasma OPG levels increase progressively throughout the course of infection, and correlate with other established markers of outcome in patients with severe malaria. Collectively, these findings suggest that OPG may have a role as a novel biomarker in patients with severe malaria infection, and also raise the intriguing possibility that OPG may play a role in modulating malaria pathogenesis.

**What is known about this topic?**

- Acute EC activation plays a critical role in the pathogenesis of *P. falciparum* malaria. This EC activation results in release of proteins stored within Weibel-Palade bodies including VWF and Ang-2.
- OPG binds to the A1 domain of VWF and consequently is also stored within the WP bodies of EC.

**What does this paper add?**

- Plasma OPG levels are markedly elevated in children with cerebral malaria.
- Plasma OPG levels correlate with clinical severity, and inversely with clinical outcome, in children with severe *P. falciparum* malaria.
- Plasma OPG levels are also markedly elevated from an early stage in a murine model of experimental CM.

## Figures and Tables

**Table 1:** **Patient characteristics and baseline data for children enrolled.** n.a. = not applicable; n.d. = not done.

	Healthy	NMFI	UM	CM
N	26	45	52	135
Age (Years)	5.7 ± 0.4	4.1 ± 0.2	5.5 ± 0.3	4.2 ± 0.1
Sex, female (%)	10 (38%)	27 (52%)	22 (49%)	64 (47%)
Mortality (%)	n.a.	n.a.	n.a.	20 (15%)
Temperature (°C)	36.6 ± 0.1	38.4 ± 0.1	38.5 ± 0.1	38.6 ± 0.1
Parasitaemia (x10^3^/µl)	n.d	n.d	117 ± 30	200 ± 25
Haemoglobin (g/dl)	9.9 ± 1.27	10.6 ± 0.2	8.8 ± 0.3	7.1 ± 0.2
Platelets (x10^3^/µl)	413.7 ± 102.9	332.5 ± 20.0	140.8 ± 12.8	102.8 ± 6.3
Lactate (mmol/l)	2.0 ± 0.1	1.8 ± 0.1	2.5 ± 0.1	6.9 ± 0.3
VWF:Ag (µg/ml)	8.2 ± 1.0	24.7 ± 1.6	15.4 ± 1.4	29.7 ± 1.2
